# Adaptive Distribution and Priority Protection of Endangered Species *Cycas balansae*

**DOI:** 10.3390/plants14050815

**Published:** 2025-03-05

**Authors:** Huayong Zhang, Yanxia Zhou, Shijia Zhang, Zhongyu Wang, Zhao Liu

**Affiliations:** 1 Research Center for Engineering Ecology and Nonlinear Science, North China Electric Power University, Beijing 102206, China; 2 Theoretical Ecology and Engineering Ecology Research Group, School of Life Sciences, Shandong University, Qingdao 250100, China; 3 Research Group WILD Department Biology, Vrije Universiteit Brussel, Pleinlaan 2, 1050 Brussels, Belgium

**Keywords:** climate change and human activities, *Cycas balansae*, MaxEnt model, adaptive distribution, priority protection areas

## Abstract

As an endangered species, the habitat of *Cycas balansae* (*C. balansae*) is subject to a variety of impacts, including climate change and human activities, and exploring its adaptive distribution and conservation areas under such conditions is crucial to protecting the ecological security of endangered species. In this study, we used the MaxEnt model and Marxan v4.0.6 to systematically evaluate the adaptive distribution and priority protection areas of the endangered species *C. balansae*. The results showed that the endangered species *C. balansae* is concentrated in Xishuangbanna and its surrounding zones in the southern Yunnan Province. The main factors affecting the distribution of *C. balansae* were temperature seasonality, mean temperature of the coldest quarter, isothermality, and precipitation of the warmest quarter, among which temperature was the dominant factor. Under different climate scenarios in the future, the adaptive distribution area of *C. balansae* showed a slight decrease, and the adaptive distribution showed a northward migration trend. The future climate distribution pattern is closely related to temperature seasonality and the mean temperature of the coldest quarter. In addition, the influence of anthropogenic disturbances on the distribution of *C. balansae* cannot be ignored. Currently, there is a large range of conservation vacancies for *C. balansae*, and it is recommended that Simao City be used as a priority conservation area. This study provides new insights for determining the priority conservation areas and conservation strategies for the endangered species *C. balansae*.

## 1. Introduction

Global warming has caused dramatic changes in the spatial distribution pattern of species, resulting in a tendency for species ranges to move towards the poles or higher altitudes and a decline in biodiversity, posing greater challenges for species conservation [1,2,3]. A large number of previous studies have shown that climate change in the future will gradually reduce the distribution area of most endangered species and gradually increase habitat fragmentation, resulting in a further increase in the risk of species extinction, especially for endangered species with small population sizes and narrow distribution ranges [4,5,6,7]. However, with global warming, human activities have also had a non-negligible impact on the natural environment. Since the Quaternary, the increasing range and intensity of human activities have directly affected the spatial distribution and diversity of species [8,9] and even led to species losing their original habitat [10,11]. The environment has, thus, gradually evolved as a result of the joint action of natural and anthropogenic processes. Therefore, modeling the appropriate distribution of species under environmental changes and determining their priority conservation areas provides a theoretical basis for the effective conservation of biodiversity in the future and is, at the same time, of great significance for maintaining the functional integrity of ecosystems.

*Cycas* are the oldest and most primitive seed plants among the existing green plants [12,13] and are known as “living fossils” [14,15]. Their origin can be traced back to the late Permian [16]. They reached their peak in the Mesozoic Jurassic dinosaur era, gradually declined in the Cretaceous period, and most cycads were extinct by the Quaternary glacial period. *Cycas* was widely distributed [17], but due to climate change and changes in geographical environment [18,19], the existing *Cycas* are only distributed in tropical and subtropical areas of Asia, Africa, Oceania, and America [20,21]. The study of cycads is of great scientific value for understanding the changes in paleoflora, paleogeography, and paleoclimate and is of great significance for the protection of biodiversity [22,23,24].

*Cycas balansae* is an evergreen shrub of *Cycas*, which is mainly distributed in the tropical rainforests or limestone monsoon rainforests in the valleys of Yunnan and Guangxi in the southern part of China, which are located at an altitude of 100–800 m and likes warm and humid environment [25], and it is also cultivated in the Sichuan, Guangdong, and Taiwan Provinces. As a species with a long geological history, it is of great significance in the study of the origin and evolution of the biodiversity of *Cycas* flora and its relationship with the environment [12]. At the same time, the roots, stems, and leaves of *C. balansae* have anti-inflammatory, analgesic, and antihypertensive effects in traditional Chinese medicine, which has certain medicinal value [26], and it has high edible and economic value because of the starch content in the pith of *C. balansae* and its beautiful tree shape [27,28]. However, *C. balansae* is a dioecious plant with a low seed-setting rate, which hinders natural expansion [15,29]. In recent years, due to the impacts of climate change and human activities, the population of *C. balansae* has declined abruptly, its distribution zone has been shrinking, and its habitat has been gradually fragmented [27]. Currently, *C. balansae* is endangered, has a limited distribution, and has been listed as an endangered species in the Red List of Biodiversity in China-Volume of Higher Plants [29]. Although studies have been conducted on the conservation of resources and microbial diversity [25,26,30], they are relatively narrow in scope and lack systematic research on the adaptive distribution and the establishment of priority conservation zones for *C. balansae*. Therefore, analyzing the impact of climate change and simulating the distribution of its suitable habitat is beneficial to the resource protection and effective utilization of this species.

In this study, we applied the data on the geographical distribution of vegetation, climate, topography, and soil. The adaptive distribution and priority protection areas for the endangered species *C. balansae* were then identified by utilizing the MaxEnt model and the Marxan v4.0.6 The specific objectives of this study are to (1) determine the adaptive distribution of *C. balansae* and the main environmental factors affecting its distribution under the current climate conditions; (2) predict and analyze future trends in the distribution and movement of *C. balansae* in suitable areas under different climate scenarios; (3) use Marxan software to determine the priority protected zones necessary for the endangered species *C. balansae*. Our study offers invaluable insights into the scientific management of and conservation strategies for *C. balansae* under the impacts of climate change.

## 2. Results

### 2.1. Adaptive Distribution and Environmental Drivers

**Figure 1:**
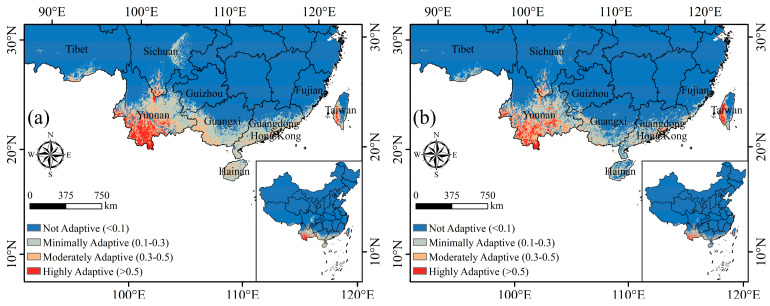
Adaptive distribution and main environmental variables of *Cycas balansae* under current climate conditions with (**b**) and without (**a**) human activities.

The adaptive distribution of the endangered species *C. balansae* is mainly concentrated in the southern region of China. Under natural environmental conditions (Figure 1a), the total adaptive distribution area of *C. balansae* only accounts for 5.40% of the total land area of the country, and the highly adaptive area accounts for 11.16% of the total adaptive distribution area, mainly distributed in the southern part of China’s Yunnan Province; while under the interference of anthropogenic activities (Figure 1b), the total area of the adaptive distribution of *C. balansae*, and the area of the minimally, moderately, and highly adaptive distribution, is lower than that of the natural environment. The proportion of the total adaptive distribution area decreased from 5.40% to 4.20%, and the area of the highly adaptive distribution decreased by 18.42%, with the decrease being especially significant in Simao City and the Xishuangbanna regions. In addition, the adaptive distribution in Guangxi and southern Guangdong, as well as Hainan and other regions, has been reduced or even lost.

**Figure 2:**
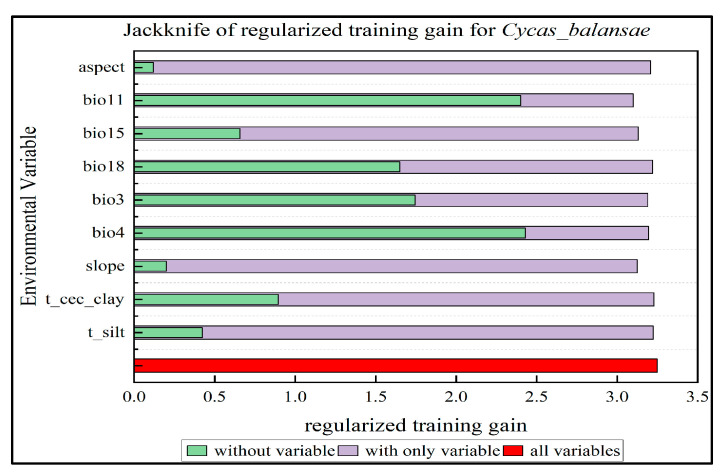
Jackknife training gain of each environmental variable for *C. balansae*. (aspect: represents the aspect; bio11: represents the mean temperature of coldest quarter; bio15: represents the precipitation seasonality; bio18: represents the precipitation of warmest quarter; bio3: represents the isothermality; bio4: represents the temperature seasonality; slope: represents the slope; t_cec_clay: represents the Topsoil CEC (clay); t_silt: represents the Topsoil silt fraction).

The results show that Figure 2 and Figure S1 temperature seasonality (bio4), mean temperature of coldest quarter (bio11), isothermality (bio3), and precipitation of warmest quarter (bio18) are the main limiting variables for the distribution of *C. balansae*, and the total contribution rate is over 90%. Among them, temperature seasonality (bio4) had the highest contribution rate, accounting for 48.7%, and, therefore, had the most obvious influence on the distribution of *C. balansae*. In addition, with the inclusion of anthropogenic factors in the modeling Figure S1B, the percentage share (7.6%) of the human activity factor’s contribution was also higher, second only to temperature and precipitation, and its influence on the distribution of *C. balansae* could not be ignored, while the influence of the rest of the variables was not significant. Overall, the distribution pattern of *C. balansae* is mainly affected by climate factors, with temperature being the most important factor affecting this distribution, followed by precipitation and human activities, while soil and topography were relatively less influential.

**Figure 3:**
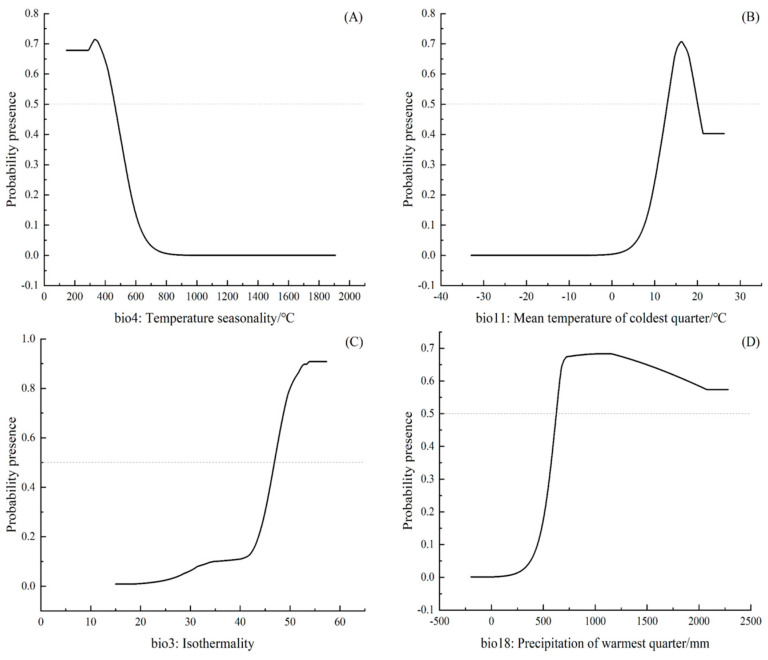
Response curves of the probability of occurrence of *C. balansae* to the temperature seasonality (**A**), the mean temperature of coldest quarter (**B**), the isotherm (**C**), and the precipitation of warmest quarter (**D**) in China.

As can be seen from Figure 3, the range of values of the optimum survival environmental variables for each of the main environmental variables affecting the distribution and growth of *C. balansae* were 172–462 °C for temperature seasonality (bio4) (Figure 3A), 13–21 °C for the mean temperature of coldest quarter (bio11) (Figure 3B), 47.5–53.5 for isothermality (bio3) (Figure 3C), and 660–2100 mm for the precipitation of the warmest quarter (bio18) (Figure 3D).

### 2.2. Adaptive Distribution Driven by Climate

**Figure 4:**
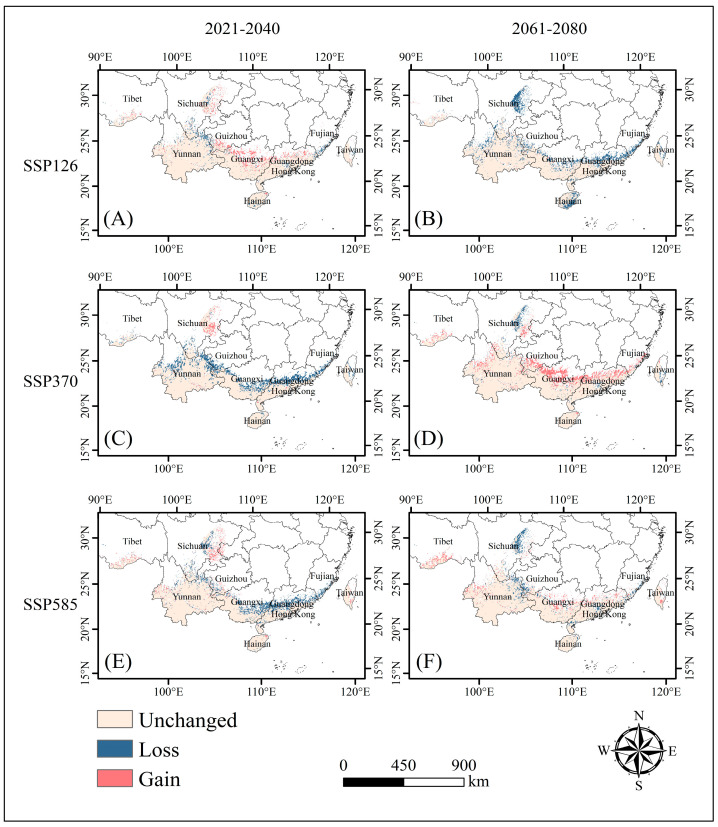
Distributions of adaptive area expansion and contraction of *C. balansae* under different future climate scenarios in comparison to the current status; “Gain” indicates areas where adaptive distribution has increased, “Loss” indicates areas where adaptive distribution has decreased, and“Unchanged” indicates areas where adaptive distribution remain unchanged. (**A**) represents the contraction and expansion of the adaptive area of *C. balansae* from the current scenario to the SSP126 scenario in 2021–2040, (**B**) represents the contraction and expansion of the adaptive area of *C. balansae* from the current scenario to the SSP126 scenario in the 2061–2080, (**C**) represents the contraction and expansion of the adaptive area of *C. balansae* from the current scenario to the SSP370 scenario in the 2021–2040, (**D**) represents the contraction and expansion of the adaptive area of *C. balansae* from the current scenario to the SSP370 scenario in 2061–2080, (**E**) represents the contraction and expansion of the adaptive area of *C. balansae* from the current scenario to the SSP585 scenario in the 2021–2040, and (**F**) represents the contraction and expansion of the adaptive area of *C. balansae* from the current scenario to the SSP585 scenario in 2061–2080.

Under future climatic conditions, the overall adaptive distribution of the endangered *C. balansae* did not change significantly compared with the current ones, and there were only slight differences in the distribution area of each level of adaptive distribution under different scenarios. The results of this study show that *C. balansae* has a distribution pattern from east to west and is mainly distributed in the southern part of Yunnan Province in the tropics and the southern part of Guangxi and Guangdong Provinces in the subtropics (Figure 4). On the whole, the adaptive distribution area of *C. balansae* showed a slightly shrinking trend, with a tendency to migrate towards northern high-latitude regions.

**Figure 5:**
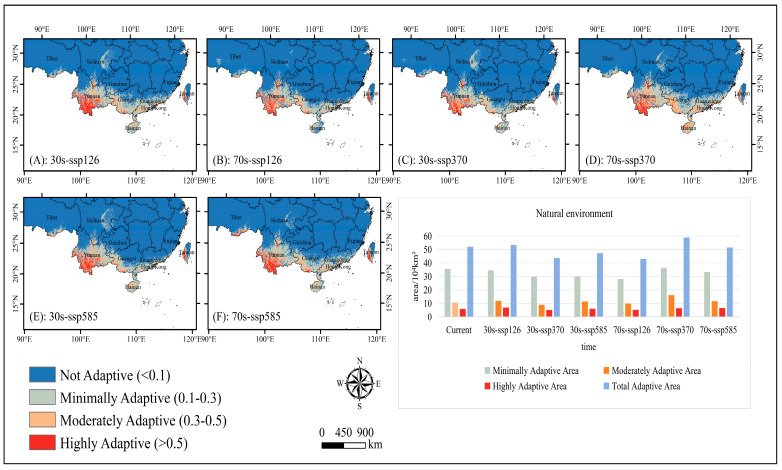
Spatial patterns and area of adaptive distribution for *C. balansae* under future climate scenarios under natural environmental conditions. (**A**) represents the adaptive distribution of SSP126 scenarios from 2021 to 2040, (**B**) represents the adaptive distribution of SSP126 scenarios from 2061 to 2080, (**C**) represents the adaptive distribution of SSP370 scenarios from 2021 to 2040, (**D**) represents the adaptive distribution of SSP370 scenarios from 2061 to 2080, (**E**) represents the adaptive distribution of SSP585 scenarios from 2021 to 2040, and (**F**) represents the adaptive distribution of SSP585 scenarios from 2061 to 2080.

Under natural environmental conditions (Figure 5), from 2021 to 2040, the adaptive distribution of *C. balansae* under different adaptive levels was similar in the SSP126 and SSP585 scenarios. Among them, the moderately and highly adaptive distribution have slightly expanded, and the minimally adaptive distribution has clearly contracted, especially in the SSP585 scenario, where its area has decreased by 15.56%. The total adaptive distribution of *C. balansae* changed the most under the SSP370 scenario compared to the SSP126 and SSP585 scenarios, as it decreased by 19.63% relative to the current situation. The adaptive distribution of minimal, moderate, and high levels showed a downward trend, and their areas decreased by 16.80%, 15.38%, and 13.60%, respectively. From 2061 to 2080, under the SSP126 scenario, the area of each adaptive distribution was reduced, with a total reduction rate of 17.40%, and the total adaptive distribution area was reduced by 19.86% compared with the 2021–2040 period. In the SSP370 scenario, the area of each adaptive distribution expanded, with a larger increase compared to all other climate scenarios, with a total growth rate of 13.04% and an increase of 25.82% compared with 2021–2040. Under the SSP585 scenario, the moderately adaptive distribution and highly adaptive distribution expanded slightly, while the minimally adaptive distribution clearly shrunk. Compared to the 2021–2040 period, the total adaptive distribution area increased by 8.06%, but it showed a downward trend as a whole.

**Figure 6:**
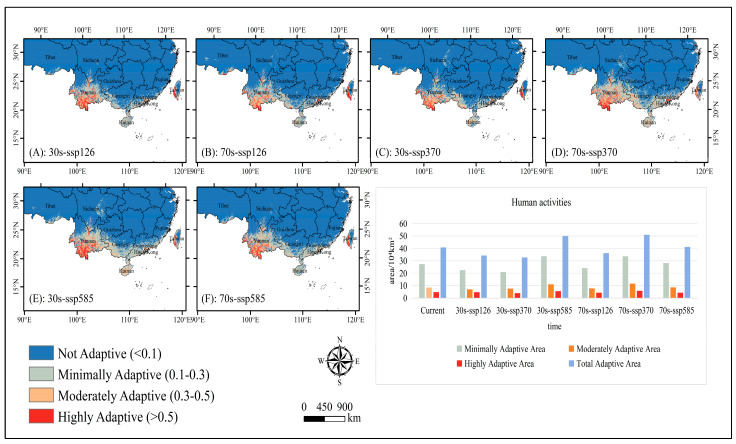
Spatial patterns and area of adaptive distribution for *C. balansae* under anthropogenic impacts for future climate scenarios. (**A**) represents the adaptive distribution of SSP126 scenarios from 2021 to 2040, (**B**) represents the adaptive distribution of SSP126 scenarios from 2061 to 2080, (**C**) represents the adaptive distribution of SSP370 scenarios from 2021 to 2040, (**D**) represents the adaptive distribution of SSP370 scenarios from 2061 to 2080, (**E**) represents the adaptive distribution of SSP585 scenarios from 2021 to 2040, and (**F**) represents the adaptive distribution of SSP585 scenarios from 2061 to 2080.

With the inclusion of anthropogenic factors in the modeling (Figure 6), the results of the study show that human activities have a greater impact on the adaptive distribution of *C. balansae*. Compared to the natural conditions, except for a slight increase in the area of the minimally adaptive distribution of *C. balansae* under the SSP585 climate scenario in 2021–2040, the fitness status of the minimally, moderately, and highly adaptive distribution under the other climate scenarios showed a decreasing trend; in particular, the total adaptive distribution of *C. balansae* shrinks the most under the SSP126 climate scenario for 2021–2040, with a total shrinkage rate of 35.84%. Meanwhile, the area of the minimally, moderately, and highly adaptive distribution shrinks by 22.77%, 8.85%, and 4.22%, respectively.

We found that the changes to *C. balansae’s* future climate distribution patterns are closely related to the temperature seasonality (bio4) and the mean temperature of the coldest quarter (bio11). These are the two main environmental factors that affect the distribution of adaptive distribution of *C. balansae* under three different climate scenarios from 2021 to 2040 and from 2061 to 2080. In order to intuitively reflect the contribution difference of climate factors in different periods and different scenarios, we removed the two factors with the highest contribution rate and found that precipitation of warmest quarter (bio18) was the main environmental variable limiting *C. balansae’s* distribution under natural conditions (Figure S2A), with contribution rates above 9%. When human activities are included (Figure S2B), precipitation of the warmest quarter (bio18) and human activities have a great influence on the distribution of *C. balansae*, especially in the three climate scenarios for the 2061 to 2080 period, where their contribution rates are higher than 8%. These results are helpful for us to better know the influence of future different climates on the suitable growth range of *C. balansae*.

### 2.3. Protection Priority Area

The results show that the stable adaptive distribution of the endangered species *C. balansae* is concentrated in the southern part of Yunnan Province, and the area suitable for planting is small. The most suitable planting area only accounts for 7.42% of the total stable adaptive distribution area. It is mainly distributed in Xishuangbanna, Simao City, and Lincang City. It is within the stable, highly adaptive distribution, and the distribution of suitable planting areas is relatively concentrated, which is beneficial to the establishment of priority protected zones.

**Figure 7:**
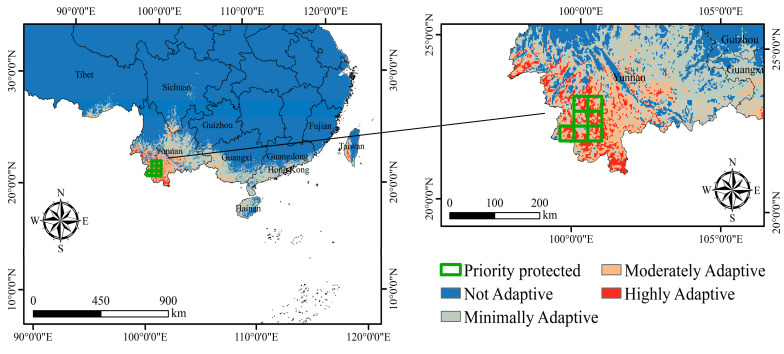
Stable adaptive distribution and priority protected areas.

The results of the study show that (Figure 7) the priority zones for protecting the endangered species of *C. balansae* are mainly concentrated in the southern part of Simao City. At present, the nature reserves of Mengla and Menghai in the Xishuangbanna region have been set up by the state for the protection of the *C. balansae* species and are not within the scope of the establishment of this protected area. In addition, Lincang City has more developed agriculture, more frequent human activities, and is more marginal in the most suitable planting area, while the distribution of the other two regions is more dispersed. Its regional cost for the establishment of a protected area is more expensive, may also have an impact on the local economy, and is not conducive to the establishment of protected areas. Simao City, on the other hand, is close to Xishuangbanna and is in the moderately to highly stable cultivation zone, and its distribution is relatively concentrated, which is conducive to the formulation of targeted protection and management. In the end, we recommend that Simao City should be designated as a priority conservation area for *C. balansae*.

## 3. Discussion

The MaxEnt model has been demonstrated to be one of the most dependable habitat modeling methods [31,32,33], which has been successfully applied to the habitat simulation of various cash crops, medicinal materials and endangered species [34,35,36,37]. It has become an important tool for suitability distribution due to its short run time, low influence of sample size, and high simulation accuracy [38,39]. It has been shown that in a small number of geographic locations (<10), even as low as 4 or 5, the MaxEnt model still produces valid and highly accurate predictions [40]. In addition, the results show that the simulation performance of the model was excellent, whether it was the study on the distribution change of *Ormosia microphylla* with 38 sample points [41], the study on the species suitability of *Horsfieldia tetratepala* with 25 sample points or the study on the potential distribution of *Ostrya rehderiana* (Betulaceae) with 15 sample points [34,42]. All the above studies further confirmed that the MaxEnt model can predict the distribution pattern of species well even if the sample size is small. In this paper, we utilized the MaxEnt model for the adaptive distribution of endangered species of *C. balansae* with an AUC value of more than 0.9 (Figure S3), indicating that the model has a high reliability and excellent simulation performance. Therefore, this model can be used to simulate the adaptive distribution of the endangered species *C. balansae*.

The relevant literature has confirmed that climatic variables are the main limiting factors determining species distribution at global, continental, or regional scales [43,44,45]. The same conclusion was reached in this study, and the results revealed that temperature seasonality (bio4), mean temperature of the coldest quarter (bio11), isothermality (bio3), and precipitation of the warmest quarter (bio18) were the main influencing variables affecting the adaptive distribution of *C. balansae*, with temperature playing a predominant role. In addition, the distribution probability dropped below 0.5 when the standard deviation of the temperature seasonality (bio4) was greater than 450 °C; the distribution probability of *C. balansae* also plummeted when the mean temperature of the coldest quarter (bio11) was below 13 °C, and it dropped down to 0 when it dropped below 0 °C, which further indicated that the growth distribution of *C. balansae* was mainly influenced by the temperature limitation. According to the literature [25,26], *C. balansae* grows naturally in a warm and humid environment, having poor cold tolerance, as too low a temperature often freezes the root system, restricts its growth, and even leads to death. Studies relevant to this case have confirmed that temperature plays a dominant role in the natural distribution of plants, as some scholars [46] found that temperature is the dominant climatic variable affecting the distribution of pests feeding on cycads. He et al. [47] found that migration, differentiation, and speciation of *Cycas* species were related to historical cooling events. The findings of each of these studies confirm the accuracy of the results of this study.

During plant growth, factors such as temperature, precipitation, and light are considered to be the most important factors affecting their distribution [48,49]. Among these factors, air temperature is the heat source for seed germination and plant growth and development, while precipitation is very important for keeping regular photochemical reactions and other metabolic transversions of plants. In this study, although temperature plays a major role in limiting the distribution of *C. balansae*, other environmental factors, such as precipitation, soil, and topography, should not be neglected as well. The ranking of contribution rates shows that the contribution of precipitation of the warmest quarter (bio18) and precipitation seasonality (bio15) accounted for 7.90% and 2.40%, respectively. In comparison, the slope factor accounted for 3.90%. The magnitude of permutation importance shows that the precipitation seasonality (bio15) was second only to the temperature seasonality (bio4) and the mean temperature of the coldest quarter (bio11), which suggests that precipitation factors and topographic factors would also have an influence on the growth and development of *C. balansae*. Related studies have shown [50,51,52] that a single type of environmental factor does not affect the growth and distribution of plants in isolation but is a process of the combined influence of multiple factors. Therefore, the potential distribution pattern of *C. balansae* in the adaptive distribution is the result of the combined influence of multiple environmental factors.

Greenhouse gas emissions will seriously affect the growth and physiological characteristics of plants, leading to significant changes in the geographical distribution pattern of most plants [53,54,55]. Thomas et al. [43] found that 15~37% of species will face the risk of extinction by 2050 under the medium emission concentration scenario, while other species will face little risk of extinction, and some species will even benefit from climate warming, which shows that the impact of climate warming on the potential geographical distribution of species has two sides. *C. balansae* is mainly distributed in southern China, belonging to tropical and subtropical distribution groups. According to the proportion of highly adaptive areas in different climate scenarios, the results show that SSP 585 > SSP 370 > SSP 126, which shows that global warming is beneficial to the growth of *C. balansae* in China to some extent. It has been shown by relevant scholars [56,57,58] that plants show an expansion trend under climate warming conditions, but this expansion is not in the long term in this study. In 2021–2040, under the SSP585 climate scenario, the total adaptive distribution area for *C. balansae* increased by 8.38% compared to SSP370, but its total adaptive distribution area decreased by 12.56% in 2061–2080 under the SSP585 climate scenario compared to the previous period (SSP370). In addition, *C. balansae* will migrate in a northern direction in different climate scenarios in the future, meaning towards high latitude and high altitude zones. Therefore, when greenhouse gas emissions exceed a certain limit, it is not conducive to species growth.

In addition to climatic factors, anthropogenic factors have become one of the major causes of changes in vegetation communities [51,59]. It has been found [60,61,62] that anthropogenic activities lead to overutilization of biological resources, which in turn leads to changes in species patterns as well as shrinkage and fragmentation of suitable habitats for species. The ranked magnitude of environmental factor contributions showed that under natural conditions, namely bio4, bio11, bio3, and bio18, contributed 48.7%, 22.3%, 10.2%, and 7.9%, respectively, while under conditions with anthropogenic activity, which included bio4, bio11, bio3, and bio18, they contributed 50.9%, 12%, 11.4%, and 9.7%, respectively. Moreover, the contribution of the human activity factor accounted for 7.60%; the size of potentially adaptive distribution areas demonstrated that the area of minimally, moderately, and highly adaptive distribution areas under different climate scenarios for *C. balansae*, except for the SSP585 climate scenario in 2021–2040, showed a decreasing trend compared to these areas under the influence of a natural environment. Both of these aspects indicate that human activities have an outstanding influence on the distribution of *C. balansae*. Some studies found that [25,26] in recent years, anthropogenic excavation and destruction, driven by economic interests, has caused the devastation of *C. balansae* habitats, resulting in its population being forced into a small piece of its past distribution and increasing the threat of extinction. Lian et al. [63] found that changes in the hotspots of the habitat of rare and endangered species in Yunnan Province are closely related to human activities. Therefore, the interference of human activities should not be ignored, and we should emphasize and increase the protection of the endangered species *C. balansae*.

Establishing protected areas is one of the best ways to protect biodiversity and maintain ecosystem balance [64], and it is the most direct and effective way to protect key, nationally protected wild plant species and their habitats [65,66,67]. Studies have shown that the endangered *C. balansae* is stable and suitable for cultivation mainly in Xishuangbanna, Simao City, and Lincang City, but the protected area for this species is relatively small, and a nature reserve has been set up only in part of Xishuangbanna, while there are still large gaps in protection in other places; thus, attention should be paid to the protection of this species, and Xishuangbanna, which is in a highly suitable habitat area, should still be used as a core protection area. In this study, we suggest that Simao City should be established as a priority reserve for conservation. The area has a subtropical monsoon climate, characterized by high temperature and humidity, with a mean annual temperature of 19 °C and about 1500 mm of annual rainfall, which is in line with the growth characteristics of *C. balansae*. In addition, the agriculture, forestry, fishery, and animal husbandry industries are more developed in Yunnan Province, so when protecting this species, we should try to protect the suitable habitat area of *C. balansae* located in this region on the basis of not harming the local economic development and the daily life of the people [64], which is in accordance with the local conditions. In addition, *C. balansae* is dioecious, and natural reproduction is inefficient. There is a lack of corresponding research on reproductive ecology, so basic research on reproduction and cultivation techniques should be vigorously carried out to build a base for the conservation of germplasm resources.

## 4. Materials and Methods

### 4.1. Data Sources and Data Processing

The administrative boundary data of China used in this study comes from the Resource and Environmental Science and Data Center of the China Academy of Sciences (http://www.resdc.cn/ accessed on 10 November 2023). The species distribution data of *C. balansae* was obtained from the databases of the China Virtual Herbarium (CVH: https://www.cvh.ac.cn/ accessed on 18 November 2023), the National Specimen Information Infrastructure (http://nsii.org.cn/2017/home.php accessed on 18 November 2023), the Chinese Field Herbarium (CFH: http://www.cfh.ac.cn/ accessed on 18 November 2023), and the Global Biodiversity Information Facility (GBIF: https://www.gbif.org/ accessed on 18 November 2023) [68], with the data collection resulting in 87 distribution points in China (Figure S4). In order to prevent the over-fitting of the model caused by the concentrated specimen distribution, the ENMTools v1.1 (https://mirrors.tuna.tsinghua.edu.cn/CRAN/web/packages/ENMTools/index.html accessed on 15 March 2024) was used to eliminate repeated distribution points, and only one effective point was reserved in the 5 km grid to ensure the modeling accuracy. Finally, 33 effective distribution data points were obtained (Table S1), which were saved in Excel in .csv format in the order of species name, longitude, and latitude. They were used to establish the MaxEnt model.

In this study, we collected 28 environmental factors such as bioclimate, soil, and topography (Table 1). The current (1970–2000) and future (2021–2040, 2061–2080) climate data are from WorldClim (https://worldclim.org accessed on 10 December 2023), have a spatial resolution of 5 km, and include 19 bioclimatic factors. The elevation data also comes from WorldClim (https://worldclim.org accessed on 10 December 2023) and has a spatial resolution of 5 km. The slope and aspect data are extracted from the elevation data by ArcGIS 10.8 ground analysis tools. The soil data consist of six soil attributes, which are derived from the World Soil Database (https://gaez.fao.org/pages/hwsd, accessed on 18 October 2023), with a spatial resolution of 1 km.

The data on human activities come from the human footprint (hf) data layer of the International Geoscience Information Network Center (https://sedac.ciesin.columbia.edu accessed on 9 September 2023). This data layer is obtained by normalizing the human impact index generated by eight global data layers, such as population density, human land use, infrastructure construction, roads, and railways, which can objectively and comprehensively reflect the strength and spatial distribution of human activities [69]. Its spatial resolution is 1 km.

Using ArcGis 10.8 software, mask extraction, cropping, resampling, and projection are carried out on all environmental factors, and the spatial resolution is adjusted to 5 km for the following study and assay. In this paper, we assume that terrain and soil variables will remain unchanged in the coming decades [51] because it is expected that climate change scenarios will not have a major influence on these factors.

A large number of studies show that the model of CMIP6 is closer to the future climate model and has a higher accuracy [70,71]. In CMIP6, four shared socio-economic paths (SSPs) were established under the future climate scenario, which are SSP126 (minimum emission), SSP 245 (moderate emission), SSP370 (medium and high emission), and SSP585 (maximum emission), which respectively represent low to high carbon emissions. The BCC-CSM2-MR climate model exploited by the Beijing Climate Center (BCC) has higher atmospheric and surface resolutions and more detailed terrain description, which makes it possible to better model and study the extreme temperature index and its trend of the global landmass, as well as the distribution of topographic precipitation and of local temperatures. It has been widely recognized in China [72] and has been used to predict endangered species because of its high reliability [73,74,75]. In this study, the future climate scenario data of SSP126, SSP370, and SSP585, with a resolution of 5 km under this climate system model, are used for research. These scenarios are analyzed in two time periods: 2021–2040 and 2061–2080.

Considering that there is some correlation between environmental variables, redundant information may be introduced, leading to over-fitting of the model [76,77], so we have eliminated some factors with strong correlations to other factors [78]. Firstly, 28 environmental variables were put into the MaxEnt software (version 3.4.4) for analysis, and variables with a contribution ratio greater than 1.0% were selected in the results to obtain the relevant environmental factors. Secondly, Pearson relativity factor (r) was analyzed by the ENMtools software for a relativity assay [79], and environmental factors with correlation coefficients higher than 0.8 and relatively small contribution rates were eliminated [80]. Finally, 9 environmental variables were used to build the MaxEnt model.

### 4.2. Model Prediction and Accuracy Assessment

The screened environmental variables and species natural distribution point data were imported into the MaxEnt model, 75% of the data points were randomly selected as the training set for validation, and the remaining 25% of the data was used as the testing set [81] for the model accuracy test. The output format was Logistic, the default model settings were used for the rest of the options, and the model was repeatedly executed 10 times.

According to the area value (AUC) under receiver operating characteristic curve (ROC), the accuracy of the model is verified, and the range of the AUC value is [0, 1]. The higher the AUC value, the better the accuracy of the prediction results [82]. It is widely accepted that when the AUC > 0.9, the prediction result is extremely accurate [83,84,85], which can reflect the potential distribution range of the species more accurately.

### 4.3. Calculation of Adaptive Distribution and Identification of Environmental Drivers

We used the spatial analysis tools in ArcGIS 10.8 to visually analyze the processed raster data. Using the natural discontinuity grading method [86], the adaptive distribution was divided into four grades: not adaptive distribution areas (0–0.1), minimally adaptive distribution areas (0.1–0.3), moderately adaptive distribution areas (0.3–0.5), and highly adaptive distribution areas (0.5–1.0), and the areas of adaptive distribution were quantified and extracted at the same time. The adaptive distribution zone of *C. balansae* was binarized using the ArcGIS 10.8 [87,88], and the expansion and contraction of *C. balansae’s* adaptive distribution were analyzed by comparing the area changes in the adaptive distribution.

The dominant environmental factors affecting the distribution of *C. balansae* were identified through a comprehensive analysis of the contribution rate of environmental factors, the importance of substitution, and the test results of the knife-cut method output by the MaxEnt model, with response intervals having a probability of existence of *p* > 0.5 being used as the selection condition [87,89]. The response curves of *C. balansae’s* dominant environmental factors were then plotted under natural conditions, and the perspectives of various environmental factors were analyzed to derive the optimum survival environmental variable value ranges of *C. balansae*.

### 4.4. Determination of Priority Protected Areas

Systematic conservation planning is an approach commonly used internationally for biodiversity conservation and for identifying priority zones for conservation [90,91]. This method achieves species conservation and minimizes conservation costs, which is important for global biodiversity conservation efforts [92,93]. With the development of research, there is more and more software for systematic conservation planning [94,95,96], such as Marxan, C-Plan, and Zonation. Currently, the Marxan model is the most widely used. Numerous studies have shown [97,98,99] that the prioritized protected zones selected by the Marxan software have higher centralization and lower human interference levels. Therefore, this model was used in this study to plan the priority protected areas for the endangered species *C. balansae*. To rationalize the establishment of protected areas, we used the fuzzy overlay tool in ArcGIS to overlay the analyses of each adaptive distribution of *C. balansae* under six current and future climate scenarios to identify stable adaptive distribution to establish cultivation planting zones; we also used the data on human activity as a unit cost to plan for the establishment of protected areas. Several studies have shown [100,101,102] that the establishment of protected areas in places with high anthropogenic intensity is economically more costly and challenging.

The specific steps of the Marxan model are as follows: (1) Planning unit and cost setting: the studied region was further divided into grid cells as planning units, and the human activity data were then used as the protection cost of each planning unit. (2) Constructing the species distribution matrix: the species distribution matrix was constructed using the stabilized *C. balansae* highly adaptive distribution. (3) Determination of the protection target: according to the protected characteristics of the target [98], the protection target of the endangered tree species *C. balansae* was set to 80%. (4) Calculation of the unit boundary length: the ArcGIS software was applied to add the plug-in ArcMarxan2.pyt to generate the data. (5) Marxan operation: the number of iterations was set to 100, the value of the boundary length modification factor (BLM) was set to 1, the other parameters were kept at their default values, and finally, the priority-protected area with the lowest protection cost and the highest spatial concentration was selected. (6) Visualization of the priority area: the results were imported into ArcGIS to produce the planning map of the priority-protected area for *C. balansae*.

## 5. Conclusions

As a first-class endangered species, the habitat of *C. balansae* is affected by various factors, such as climate change and human activities, and the natural resources are facing depletion. This study, based on the species distribution data, climate data, soil data, and topographic data, used the MaxEnt model and the Marxan software to systematically study the adaptive distribution and priority conservation areas of this species. The results showed that (1) the adaptive distribution of *C. balansae* is mainly distributed in Xishuangbanna and its surrounding areas in southern Yunnan Province. Temperature seasonality, mean temperature of the coldest quarter, isothermality, and precipitation of the warmest quarter were the main influencing factors on the distribution of this species. In addition, anthropogenic disturbances had a large impact on the distribution of *C. balansae*, with a contribution of 7.9% according to the participatory modeling, and the total habitable area decreased by 21.91% compared with natural environmental conditions. (2) Under the influence of global climate change, the adaptive distribution area of *C. balansae* shows a slight trend of shrinking, and the adaptive distribution tends to migrate to more northern latitudes. Amongst different climatic scenarios for the future, the SSP370 scenario is more favorable to the growth of this species, and the adaptive growth area of each adaptive level shows a trend of expansion. On the contrary, the area changes of the SSP126 and SSP585 climate scenarios are more fluctuating. In addition to the temperature seasonality and the mean temperature of the coldest quarter, the distribution pattern of *C. balansae* is strongly influenced by the precipitation of the warmest quarter and human activity factors. (3) Cultivation zones of *C. balansae* in stable adaptive distribution are mainly located in Xishuangbanna, Simao City, and Lincang City; in order to ensure the survival of this species, and taking into account the economic costs and local social development, etc., we propose to set up a priority protected area in Simao City, and to strengthen in-depth investigations and monitoring of suitable regions not reported to be related to *C. balansae* (Hainan, Taiwan, and Sichuan). By systematically understanding the adaptive distribution and the factors affecting them and implementing priority conservation measures for them, we can provide valuable scientific guidance for conservation planning and for promoting the sustainable development and biodiversity conservation of *C. balansae*.

## Figures and Tables

**Table 1:** Initial environmental variable.

Code	Variables Name	Code	Variables Name
Bio1	Annual mean temperature	Bio15	Precipitation seasonality
Bio2	Mean diurnal range	Bio16	Precipitation of wettest quarter
Bio3	Isothermality	Bio17	Precipitation of driest quarter
Bio4	Temperature seasonality	Bio18	Precipitation of warmest quarter
Bio5	Max temperature of warmest month	Bio19	Precipitation of coldest quarter
Bio6	Min temperature of coldest month	ele	Elevation
Bio7	Temperature annual range	slo	Slope
Bio8	Mean temperature of wettest quarter	asp	Aspect
Bio9	Mean temperature of driest quarter	T_SILT	Topsoil silt fraction
Bio10	Mean temperature of warmest quarter	T_SAND	Topsoil sand fraction
Bio11	Mean temperature of coldest quarter	T_CLAY	Topsoil clay fraction
Bio12	Annual precipitation	T_pH_H2O	Topsoil pH (H2O)
Bio13	Precipitation of wettest month	T_ESP	Topsoil sodicity (ESP)
Bio14	Precipitation of driest month	T_CEC_CLAY	Topsoil CEC (clay)

## Data Availability

All links to input data are reported in the manuscript and all output data are available upon request to the authors.

## Supplementary Materials

The following supporting information can be downloaded at: https://www.mdpi.com/article/10.3390/plants14050815/s1, Figure S1: The contribution rate and importance of the main environmental variables of *Cycas Balansae* under the current climatic conditions with (B) and without (A) human activities; Figure S2: Contribution rate of main environmental impact factors of *Cycas Balansae* with (B) and without (A) human activities in future climate scenarios; 30s-ssp126: represents the climate scenario of SSP126 in 2021-2040, 30s-ssp370: represents the climate scenario of SSP370 in 2021-2040, 30s-ssp585: represents the climate scenario of SSP585 in 2021-2040, 70s-ssp126: represents the climate scenario of SSP126 in 2061-2080, 70s-ssp370: represents the climate scenario of SSP370 in 2061-2080, and 70s-ssp585: represents the climate scenario of SSP585 in 2061-2080; Figure S3: Receiver operating characteristic (ROC) curve. The values shown are the average of 10 replications; Figure S4: Distributions on occurrence points of *Cycas Balansae* edulis in China; Table S1: The geographic coordinates used to generate the potential distribution models of *Cycas Balansae*.
