# Supplementary material for: Adaptive Distribution and Priority Protection of Endangered Species Cycas balansae
Source: Plants (Basel). 2025 Mar 5;14(5):815. doi: 10.3390/plants14050815 (PMC11902338; doi:10.3390/plants14050815)
Supplement: Supplementary file 1 [file plants-14-00815-s001.zip › plants-3357846-supplementary.pdf]

Supplementary Information

Adaptive Distribution and Priority Protection of Endangered Species *Cycas balansae*

Huayong Zhang [1,2,*], Yanxia Zhou [1], Shijia Zhang [3], Zhongyu Wang [1] and Zhao Liu [2]

1 Research Center for Engineering Ecology and Nonlinear Science, North China Electric Power University, Beijing 102206, China
2 Theoretical Ecology and Engineering Ecology Research Group, School of Life Sciences, Shandong University, Qingdao 250100, China
3 Research Group WILD Department Biology, Vrije Universiteit Brussel, Pleinlaan 2, 1050 Brussels, Belgium
* Correspondence: zhanghuayong@sdu.edu.cn

This word file includes

Figure and Table Legends

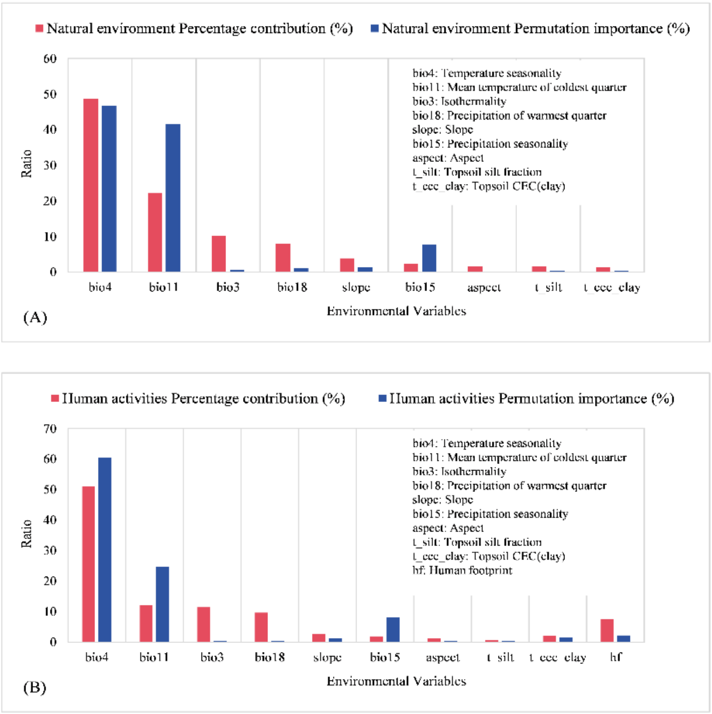

Figure S1. The contribution rate and importance of the main environmental variables of *Cycas Balansae* under the current climatic conditions with(B) and without (A) human activities.

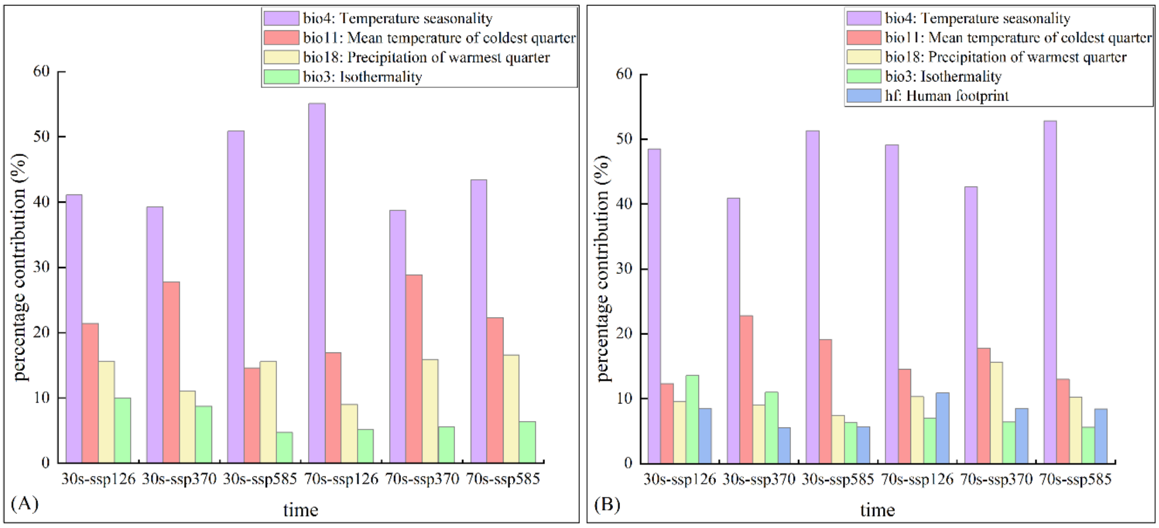

Figure S2. Contribution rate of main environmental impact factors of *Cycas Balansae* with(B) and without(A) human activities in future climate scenarios; 30s-ssp126: represents the climate scenario of SSP126 in 2021-2040, 30s-ssp370: represents the climate scenario of SSP370 in 2021-2040, 30s-ssp585: represents the climate scenario of SSP585 in 2021-2040, 70s-ssp126: represents the climate scenario of SSP126 in 2061-2080, 70s-ssp370: represents the climate scenario of SSP370 in 2061-2080, and 70s-ssp585: represents the climate scenario of SSP585 in 2061-2080.

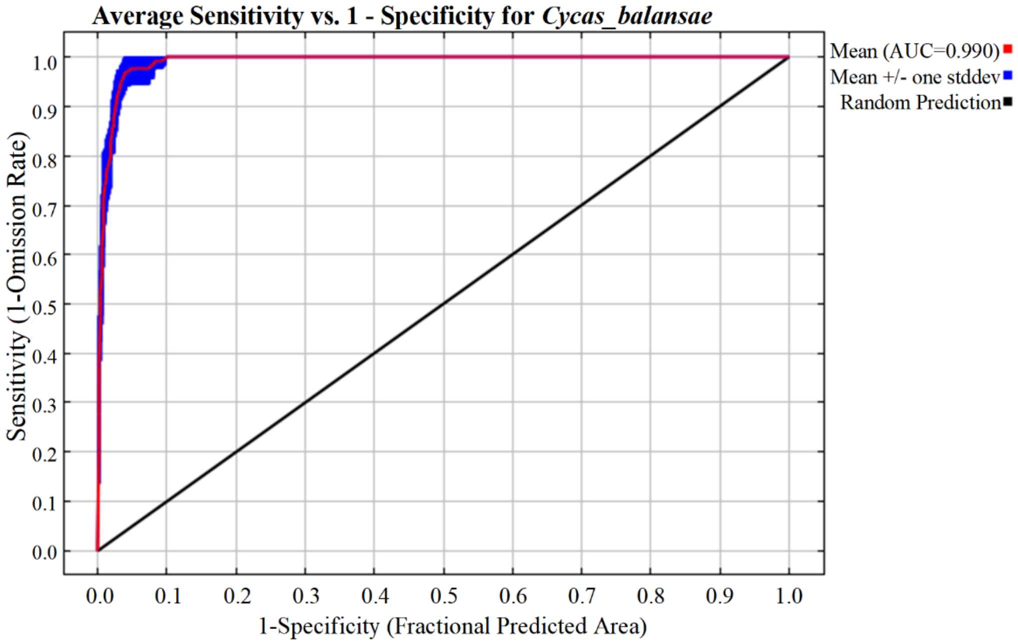

Figure S3. Receiver operating characteristic (ROC) curve. The values shown are the average of 10 replications.

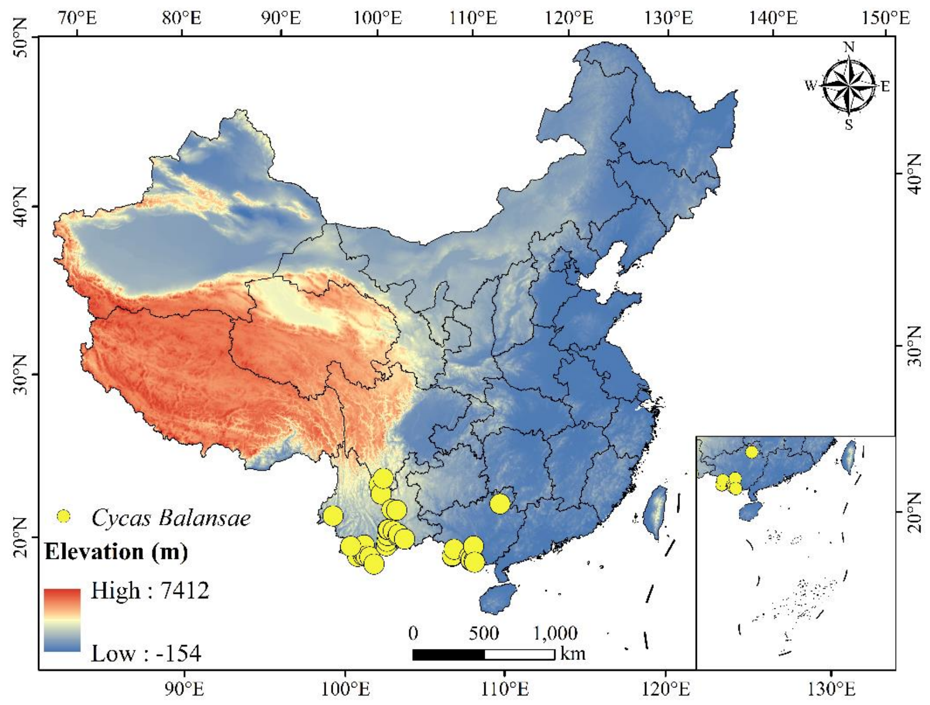

Figure S4. Distributions on occurrence points of *Cycas Balansae* edulis in China.

Table S1. The geographic coordinates used to generate the potential distribution models of *Cycas Balansae.*

Species	Longitude	Latitude
Cycas balansae	100.44	21.94
Cycas balansae	100.81	22.02
Cycas balansae	100.85	22.19
Cycas balansae	100.85	22.69
Cycas balansae	101.02	21.98
Cycas balansae	101.27	21.94
Cycas balansae	101.56	21.48
Cycas balansae	101.73	26.56
Cycas balansae	101.90	25.98
Cycas balansae	102.02	26.98
Cycas balansae	102.35	22.69
Cycas balansae	102.40	22.98
Cycas balansae	102.44	23.35
Cycas balansae	102.44	23.73

Cycas balansae	102.48	23.73
Cycas balansae	102.69	25.02
Cycas balansae	102.81	23.65
Cycas balansae	103.02	24.98
Cycas balansae	103.15	23.35
Cycas balansae	103.19	23.44
Cycas balansae	103.60	23.15
Cycas balansae	106.85	22.02
Cycas balansae	106.85	22.06
Cycas balansae	106.98	22.48
Cycas balansae	108.02	21.98
Cycas balansae	108.10	21.73
Cycas balansae	108.10	21.77
Cycas balansae	108.31	21.69
Cycas balansae	108.31	22.65
Cycas balansae	108.35	21.60
Cycas balansae	110.27	25.27
Cycas balansae	98.60	24.44
